# Trans-hiatal small-bowel herniation after laparoscopic radical total gastrectomy for gastric cancer: a case report and literature review

**DOI:** 10.1093/jscr/rjag776

**Published:** 2026-09-30

**Authors:** Dalai Xu, Lei Qiu, Feng Lu, Xuzhu Gao, Yongchang Miao, Gang Wang

**Affiliations:** Department of General Surgery, The Second People's Hospital of Lianyungang, No. 41 Hailian East Road, Haizhou District, Lianyungang 222006, Jiangsu, China; Department of General Surgery, The Second People's Hospital of Lianyungang, No. 41 Hailian East Road, Haizhou District, Lianyungang 222006, Jiangsu, China; Department of General Surgery, The Second People's Hospital of Lianyungang, No. 41 Hailian East Road, Haizhou District, Lianyungang 222006, Jiangsu, China; Clinical Oncology Research Institute, The Second People's Hospital of Lianyungang (Lianyungang Cancer Hospital), No. 161 Xingfu South Road, Haizhou District, Lianyungang 222023, Jiangsu, China; Department of General Surgery, The Second People's Hospital of Lianyungang, No. 41 Hailian East Road, Haizhou District, Lianyungang 222006, Jiangsu, China; Department of General Surgery, The Second People's Hospital of Lianyungang, No. 41 Hailian East Road, Haizhou District, Lianyungang 222006, Jiangsu, China

**Keywords:** gastric cancer, cardia cancer, laparoscopic total gastrectomy, Roux-en-Y reconstruction, hiatal hernia, diaphragmatic hernia, postoperative complication

## Abstract

Trans-hiatal hernia is a rare but serious complication after total gastrectomy. We report a 71-year-old man who underwent laparoscopic radical total gastrectomy, D2 lymphadenectomy, and Roux-en-Y reconstruction for cardia adenocarcinoma. Computed tomography (CT) on postoperative day 17 showed intrathoracic displacement of the esophagojejunal anastomosis without obstruction. Ten months later, he developed positional upper abdominal pain, and CT revealed herniation of the anastomosis and a dilated jejunal loop through the esophageal hiatus into the right thoracic cavity. Emergency laparoscopic reduction and hiatal repair were performed. The bowel was viable, and crural, and pleural defects were repaired with sutures. Recovery was uneventful. Early postoperative intrathoracic migration of the anastomosis may be a warning sign of delayed trans-hiatal hernia, and symptomatic patients require prompt CT diagnosis and surgical repair.

## Introduction

Laparoscopic radical gastrectomy is widely used for resectable gastric cancer [1–5]. Reduced adhesion formation after minimally invasive surgery may make internal hernia-related complications more apparent. After total gastrectomy with Roux-en-Y reconstruction, Petersen’s hernia and jejunal mesenteric defect hernia are better recognized, whereas trans-hiatal hernia remains rare [6–9]. Symptoms are nonspecific and may mimic obstruction, anastomotic problems, cardiopulmonary disease, or recurrence. We report a case of trans-hiatal small-bowel herniation after laparoscopic radical total gastrectomy, emphasizing an early computed tomography (CT) clue and emergency laparoscopic management.

## Case presentation

A 71-year-old man with a body mass index of 23.4 kg/m^2^ underwent gastroscopy for upper abdominal discomfort on 9 March 2023. Biopsy confirmed cardia adenocarcinoma. Preoperative CT showed no pleural abnormality, diaphragmatic discontinuity, or distant metastasis (Fig. 1a and b). The tumor was Siewert type II. He had no previous abdominal surgery, chronic cough, constipation, recurrent vomiting, smoking history, or other clear factor increasing intra-abdominal pressure.

**Figure 1:**
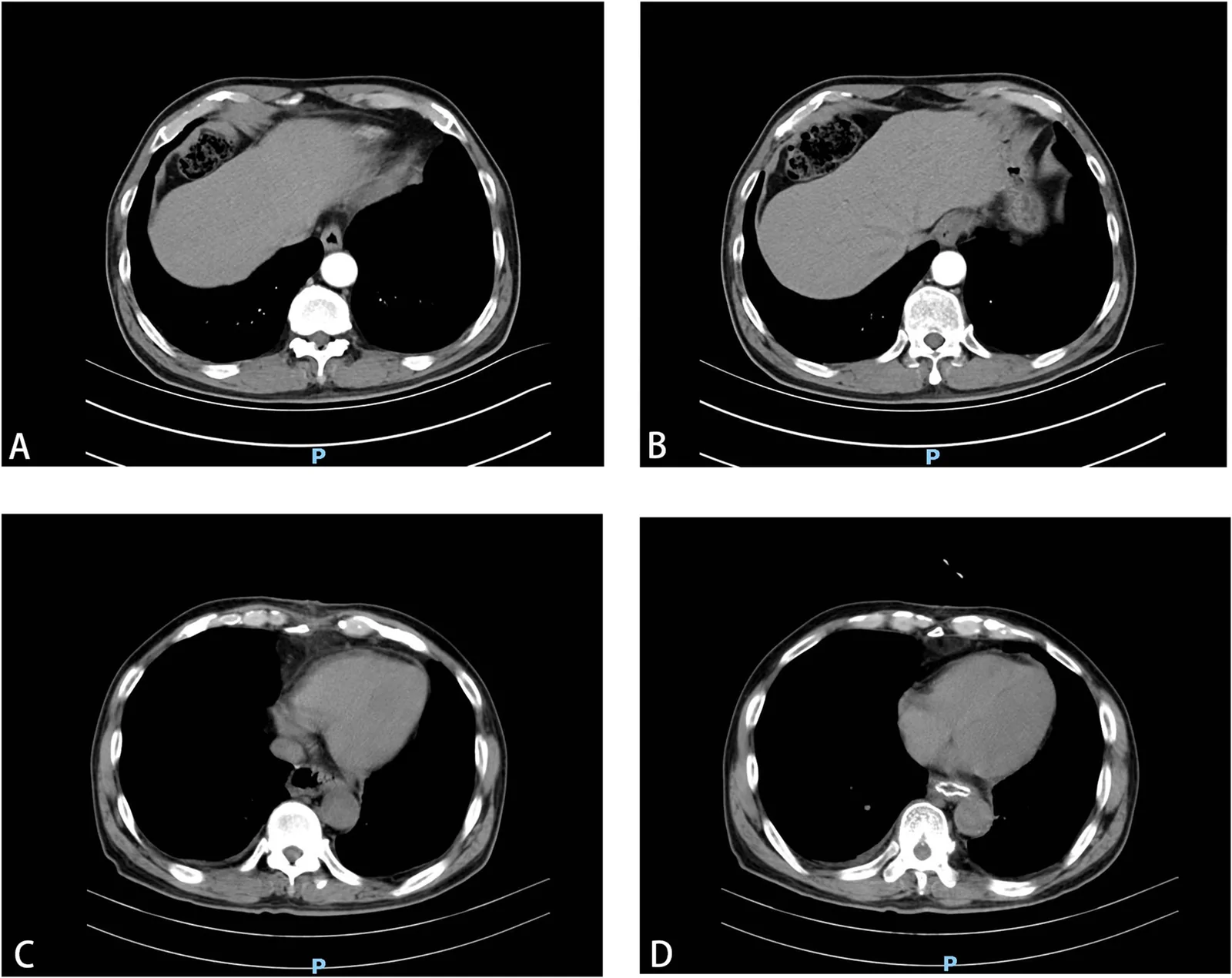
Chest and abdominal CT findings before and after laparoscopic radical total gastrectomy. (A and B) Preoperative CT showed intact bilateral pleura, without obvious pleural thickening, pleural effusion, or interruption of diaphragmatic continuity. (C and D) CT on postoperative day 17 showed that the esophagojejunal anastomosis was located within the thoracic cavity, with localized pleural thickening and a small right pleural effusion.

On 26 March 2023, he underwent laparoscopic radical total gastrectomy, D2 lymphadenectomy, and Roux-en-Y reconstruction. The lower mediastinum was not entered, the esophageal hiatus was not intentionally enlarged, and no pleural injury was recognized. The esophagojejunal anastomosis used the overlap method, the Roux limb was antecolic, and the Petersen and jejunal mesenteric defects were closed. Pathology showed moderately to poorly differentiated gastric adenocarcinoma, pT3N0M0, with 0/22 positive lymph nodes and negative margins.

On postoperative day 17, CT showed localized pleural thickening, a small right pleural effusion, and intrathoracic displacement of the esophagojejunal anastomosis (Fig. 1c and d). There was no obstruction or ischemia, and the patient was discharged after stabilization. He later received adjuvant chemotherapy without recurrent vomiting, cough, constipation, poor intake, or weight loss.

About 10 months after surgery, he presented with positional upper abdominal pain. Vital signs were stable, and cardiac evaluation was unremarkable. CT showed herniation of the esophagojejunal anastomosis and a dilated jejunal loop through the esophageal hiatus into the right thoracic cavity (Fig. 2).

**Figure 2:**
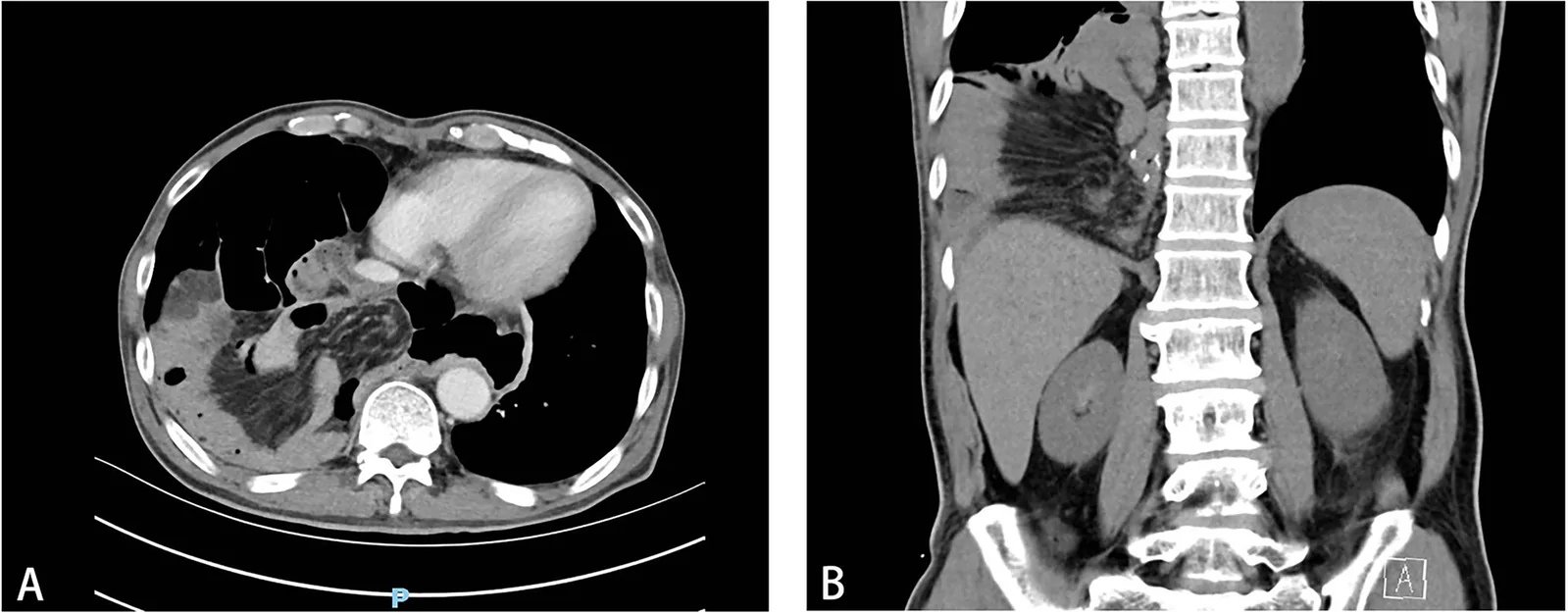
Emergency chest and abdominal CT findings 10 months after the initial operation. (A) Axial image showing herniation of part of the small bowel and the esophagojejunal anastomosis through the esophageal hiatus into the right thoracic cavity. (B) Sagittal image showing dilated bowel within the thoracic cavity compressing the right lung.

Emergency laparoscopy revealed small bowel herniating through the esophageal hiatus. No recurrence or metastasis was seen. The bowel was reduced and remained viable, so resection was unnecessary. A crural muscular defect and absent pleural covering were identified. The hiatus was repaired using 3-0 barbed and 3-0 nonabsorbable sutures (Fig. 3). He passed flatus on postoperative day 2 and was discharged on day 5. At 3 months, he had no abdominal or respiratory symptoms.

**Figure 3:**
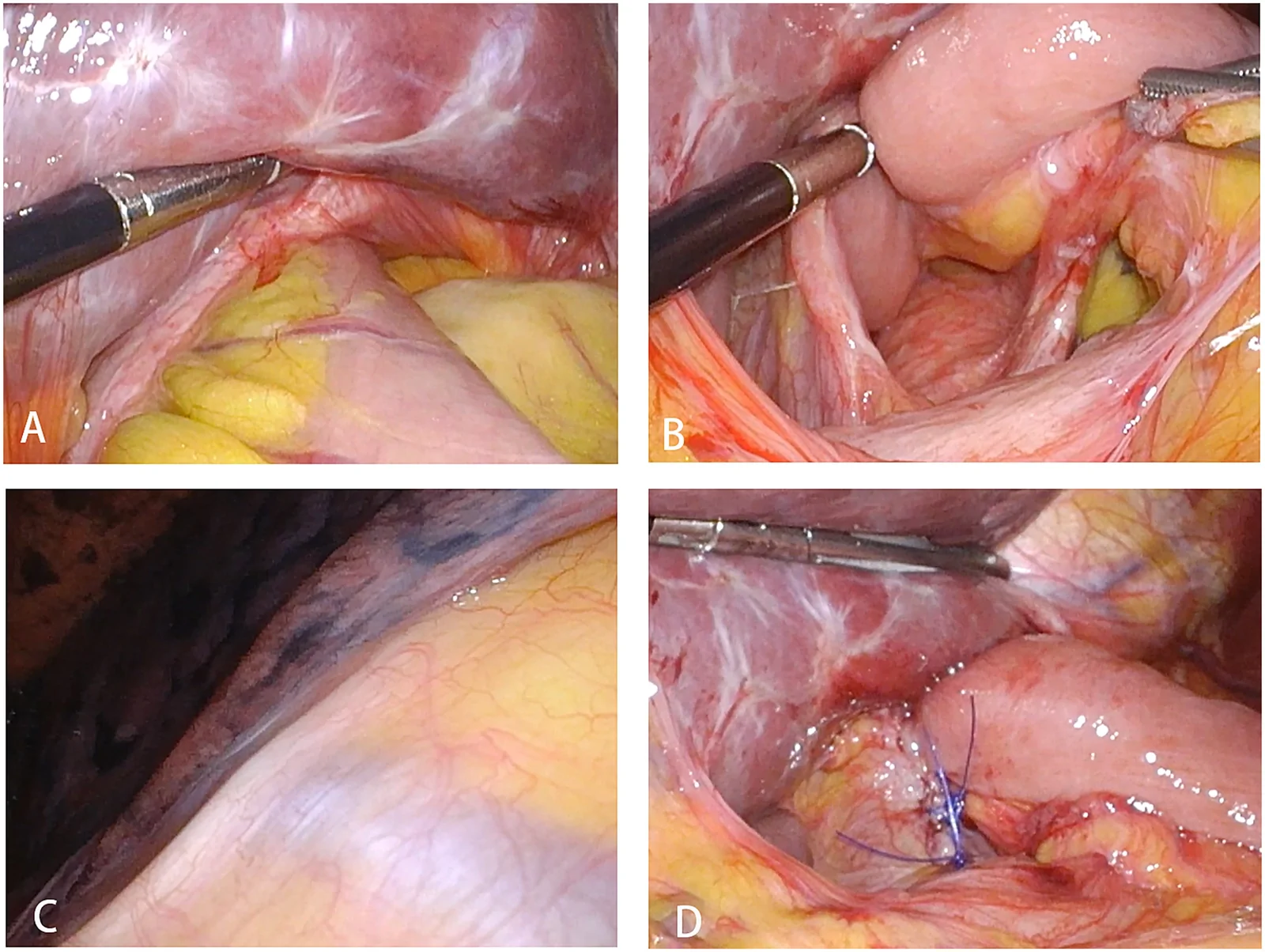
Intraoperative findings during emergency laparoscopic exploration and hiatal hernia repair. (A) The small bowel herniated into the thoracic cavity was reduced using atraumatic laparoscopic forceps. (B) A muscular defect in the region of the diaphragmatic crura was visible after reduction. (C) Exploration through the esophageal hiatus toward the thoracic cavity showed no intact pleural covering, indicating a pleural defect. (D) The esophageal hiatus and crural defect were repaired using 3-0 barbed suture combined with 3-0 nonabsorbable suture.

## Discussion

Trans-hiatal hernia after total gastrectomy differs from ordinary sliding hiatal hernia because the herniated contents are usually small bowel, transverse colon, or omentum [7–13]. Contrast-enhanced CT is preferred because it shows the herniated contents, hiatal route, bowel dilatation, and ischemic signs [8–12]. In this case, postoperative day 17 CT already showed intrathoracic displacement of the esophagojejunal anastomosis. This may have reflected early hiatal weakness, anastomotic migration, or occult pleural/diaphragmatic injury. Similar findings should prompt multiplanar CT review, patient education, and closer follow-up.

The mechanism was likely multifactorial. Although no pleural injury or hiatal enlargement was recorded, later surgery showed a crural and pleural defect. Occult microinjury or secondary tissue failure after traction, edema, ischemia, or impaired healing may have contributed. Reduced adhesions after laparoscopy and mobility of an antecolic Roux limb may have allowed gradual migration under negative intrathoracic pressure [6, 7, 11–14]. Causality cannot be proven from one case.

Symptomatic trans-hiatal hernia after total gastrectomy should be treated promptly, especially when CT shows dilatation, thoracic compression, or suspected incarceration. Delay may cause ischemia, necrosis, or perforation [8–12]. Laparoscopy is feasible in stable patients without severe distension or contamination. Prevention requires careful preservation of the crura and pleura, avoidance of unnecessary hiatal enlargement, reassessment after reconstruction, closure of mesenteric defects, and immediate repair of crural or pleural injury [9, 13].

## Conclusion

Trans-hiatal small-bowel herniation is a rare but serious complication after laparoscopic radical total gastrectomy. Positional upper abdominal pain should prompt contrast-enhanced chest and abdominal CT. Early intrathoracic displacement of the esophagojejunal anastomosis may be a warning sign. Symptomatic patients require early reduction, hiatal repair, and follow-up.

## Ethics approval and consent to participate/publish

Written informed consent was obtained from the patient before submission of this case report, including consent for publication of clinical information, imaging findings, and intraoperative photographs. This study was approved by the Ethics Committee of Lianyungang Second People’s Hospital (approval No. 2021-019-01).
